# PBL triggers in relation to students’ generated learning issues and predetermined faculty objectives: Study in a Malaysian public university

**DOI:** 10.12669/pjms.322.9248

**Published:** 2016

**Authors:** Nurul Hidayati Ruslai, Abdus Salam

**Affiliations:** 1 Nurul Hidayati Ruslai, BSc Hons, MMedEd. Lecturer, Department of Biology, Centre for Foundation Studies, International Islamic University of Malaysia (IIUM), Petaling Jaya, Malaysia; 2 Abdus Salam, MBBS, MPH, PgDipMedEd, MMedEd. Associate Professor, Medical Education Department, Faculty of Medicine, Universiti Kebangsaan Malaysia (UKM) Medical Centre, (The National University of Malaysia), Kuala Lumpur, Malaysia

**Keywords:** PBL triggers, Learning issues, Faculty objectives, Similarities, Training needs, Organizational development

## Abstract

**Objectives:**

Foundational elements of problem based learning (PBL) are triggers, tutors and students. Ineffective triggers are important issues for students’ inability to generate appropriate learning issues. The objective of this study was to evaluate PBL triggers and to determine similarities of students’ generated learning issues with predetermined faculty objectives.

**Methods:**

It was a retrospective study conducted in 2014 analyzing all 24 PBL-triggers used at Centre for Foundation Studies, International Islamic University Malaysia, in four semesters during two consecutive years 2011 and 2012. Triggers were used as textual and illustration format equally in each semester. Total 16 PBL-triggers with highest and lowest achieving similarities of learning issues with predetermined faculty objectives were selected equally from each semester and format. The trigger quality and learning issues related to predetermine faculty objectives were analyzed and presented as mean and percent distribution.

**Results:**

Mean similarities score of students’ generated learning issues were 3.4 over 5 predetermined faculty objectives which was 68%, varied from 58% to 79%. More than 70% similarities were generated from five textual and four illustrated triggers, while <70% similarities observed from four illustrated and three textual triggers.

**Conclusion:**

Whatever the trigger formats in PBL, it is the designing considering influential variables that influence higher outcomes. Triggers should have planned clues that lead students to generate issues correlate with faculty objectives. Educational institution should emphasize on training needs of faculty at regular interval to develop and re-in force teachers’ skills in trigger design, thereby to promote a sustainable educational and organizational development.

## INTRODUCTION

Problem based learning (PBL) is an innovative student-centered strategy that encourages learning through enquiry and exploration. Learning here results from process of working towards understanding a problem which acts as a stimulus for development of analytical and self-directed learning skills.^1^

Centre for Foundation Studies, International Islamic University of Malaysia (CFS IIUM) take initiative to inculcate PBL as one of the instructional strategies in their Biology module. The CFS IIUM is a centre that offered pre-university courses to Sijil Pelajaran Malaysia (SPM), equivalent to “O” level certificate. Sciences and arts courses are offered in CFS IIUM, where 400 students are admitted annually. Subjects taught in science courses are Biology, Chemistry, Physics and Mathematics. PBL has been implemented to teach Biology since June 2011.

The three foundational elements in PBL are triggers or problems, students, and tutors which closely interacts with each other.^2^ The problems quality has direct influence on PBL process and outcomes.^3^ Problems are typically a set of descriptions of situations in need of explanations and resolution used to contextualize real-world scenarios. Problems are classified as explanation, description, or strategic based on objectives and are often presented in textual format, sometimes as illustrations, pictures, videos and simulations.^4^ Effective problems are designed with well-mapped educational concepts and strategic clues, thereby lead students to generate learning issues co-related with predetermined faculty objectives.^5^ How well faculty objectives are addressed by students is a concern in PBL for both faculty and students.^6^ The objectives of this study was to evaluate PBL triggers used at CFS IIUM in order to explore similarities of students’ generated learning issues with predetermined faculties’ objectives aimed at educational and eventually organizational development.

## METHODS

It was a retrospective study which investigated the PBL triggers during 2014 under project agreement between Universiti Kebangsaan Malaysia (UKM) and IIUM. The first author was a lecturer of Biology at CFS IIUM, who conducted this study as partial fulfillments for the degree of her Master in Medical Education under the direct supervision of second author at Medical Education Department, UKM Medical Centre, Malaysia.

At the department of Biology in CFS IIUM, 400 students registered yearly from six foundation programs such as pre-medical, pre-dentistry, pre-pharmacy, pre-nursing, pre-biological and pre-allied health sciences. Students were divided into 16 large group consisting of 25 students in each group, which again sub-divided into six small sub-groups comprising of 4-5 students for PBL. Biology was taught through 12 topics in each semester and designated as Biology-I in semester-I and Biology-II in semester-II. Among 12 topics, six were covered through traditional teaching methods and six were taught through PBL. Of six PBL, three used textual and other three illustrated formatted triggers equally in each semester.

Study populations were all 24 PBL triggers used during 1^st^ and 2^nd^ semester of year 2011 and 2012. Students discussed six PBL triggers during each semester in small sub-groups and generated learning issues which were recorded in PBL log sheet used by CFS IIUM. PBL log sheet is a kind of format composed of i) facts –where information extracted from triggers, ii) ideas –where hypothesized, iii) learning issues –where questions identified, and iv) actions –where activities considered necessary to solve the questions. One large group of students produced one PBL log sheet per trigger. On an average, there were five predetermined faculty objectives against each trigger. For this study, two textual and two illustrated formatted triggers were chosen from each semester based on their highest and lowest similarities of learning issues generated by students. So, from four semesters, a sample of 4x4=16 triggers was selected purposively. Student generated learning issues in groups were collected by researchers from 16 respective lecturers of Biology. Trigger quality and similarities between students’ generated learning issues and pre-determined faculty objectives against the sampled triggers were analyzed and presented as mean and percentage distribution.

## RESULTS

Distribution of semester, year and module wise PBL topics, trigger formats, predetermined faculty objectives and its similarities with students’ generated learning issues as mean ± SD and percentage are shown in Table-I. The similarities varied from 59%-75% in semester-I Biology-I, during 2011 and 58%-73% in semester-II Biology-II during 2011. While in 2012, it was 62%-77% in semester-I Biology-I and 58%-79% semester-II Biology-II.

**Table-I:** Semester, year, and module wise PBL topics, trigger formats, number of faculty objectives and mean ± SD with percentage of similarities of students’ generated learning issues with predetermined faculty objectives against 16 PBL triggers.

Semester/Year/Module	PBL Topics	Trigger Formats	Faculty objectives	Similarities of students’ generated learning issues	

				Mean ±SD	%
Semester-I/2011/Biology-I	1. Membrane transport	Textual	4	3±0.73	75.0
	2. Enzyme	Illustration	5	3.63±0.5	72.5
	3. Variation	Textual	6	3.63±0.72	60.4
	4. Biological compound	Illustration	6	3.56±0.96	59.4
Semester-II/2011/Biology-II	5. Homeostasis	Illustration	4	2.94±0.85	73.4
	6. Reproduction	Textual	5	3.56±0.63	71.3
	7. Biotechnology	Textual	6	3.88±0.81	64.6
	8. Immunology	Illustration	6	3.5±0.63	58.3
Semester-I/2012/Biology-I	9. Ultra structure of cell	Illustration	4	3.69±0.79	76.6
	10. Cell cycle	Textual	4	2.88±0.89	71.9
	11. DNA and protein synthesis	Textual	5	3.31±0.48	66.3
	12. Photosynthesis	Illustration	6	3.69±0.79	61.5
Semester-II/2012/Biology-II	13. Hormones	Illustration	5	3.94±0.77	78.8
	14. Locomotion	Textual	4	3.06±0.85	76.6
	15. Quantitative ecology	Textual	5	3.5±0.63	70.0
	16. Development	Illustration	6	3.5±0.89	58.3

Characteristics of PBL triggers with 79% highest and 58% lowest similarities between students’ generated learning issues and predetermined faculty objectives, where trigger formats were illustrated in both situations are shown in Table-II.

**Table-II:** Characteristics of PBL triggers having highest and lowest similarities between students’ generated learning issues and predetermined faculty objectives n=8.

PBL Topics	PBL trigger characteristics in brief	% of similarities
13. Hormones	Illustrated: A figure of two groups of evening primrose flowers with two different treatments light and dark period and asked to explain the flowering responses of treatment as well as to identify type of plant for photoperiodic control of flowering response.	78.8
9. Ultra structure of cell	Illustrated: A picture entitled ‘The endomembrane system in an animal cell” labeled with 4 organelles as Q, R, S, T and asked on their abundant in the cell that synthesize steroid hormone from cholesterol and process involved in the flow of proteins.	76.6
1. Membrane transport	Textual: Rahman, who suffered from fatal hyponatremia (low sodium concentration in the body fluids) died immediately after consuming excessive amounts of water. You should examine the tonicity of the extracellular environment created by water consumption, effect on cells and impacts on different tissues/organs in the body.	75.0
5. Homeostasis	Illustrated: A graph representing effect of external temperature on body temperature of an organism and asked what happen to the body temperature when external temperature increases from 15º to 40 º C and to provide physiological responses occur in organism.	73.4
12. Photo synthesis	Illustrated: Two pictures showing carbon dioxide fixation pathways as Q and R and questioned on how they differ, how many molecules of carbon dioxide needed to synthesize two molecules of glucose, whether the Malate produces in Q and R are similar or different, explain why.	61.5
4. Biological compound	Illustrated: Structures of three biological molecules as I, II and III and asked, which of these present in animal cell, what makes them differ, what polymer produced when structure I undergo a dehydration process.	59.4
8. Immunology	Illustrated: A flow chart of local inflammatory response due to a deep punctured wound caused by stepping on a rusty nail revealed cell P releases substance Q that causes event X leads to increase temperature and event Y leads to phagocytes migrated to site of injury and asked some questions.	58.3
16. Development	Illustrated: A picture of uterus showing events from ovulation until implementation in human development and asked about process that prevents entry of additional sperms as only one sperm can fertilize the eggs, results if the cells at stage X becomes separated and consequences if Y cell fails to secrete this hormone.	58.3

## DISCUSSION

Overall mean similarities of students’ generated learning issues in the present study was 3.4 besides an average 5 (4-6) predetermined faculty objectives, which is 68% in average, varying from 58% to 79%. Dolmans et al.^7^ studied among 120 second year students assigned in 12 groups given same 12 problems with pre-identified 51 objectives, reported 64.2% similarities in average, which is close to our study findings. Van Gessel et al.^8^ and O’Neill^9^ also found 62% similarities, however for individual problems, it varied from 27.7%-100%, while in our study it is 58%-79%. Current findings support the understanding of skills of effective trigger design by lecturers at CFS IIUM. Although current findings are consistent with other studies, low matched triggers need to be examined further. Variation in generating learning issues by students also depends on facilitation skills, group dynamics and students’ prior-knowledge.^6^ Ineffective triggers makes it difficult for students to generate accurate learning issues, thus lacked in content coverage.^7^

Higher similarities of students’ generated learning issues were observed against fewer number of predetermined faculty objectives (Table-I). Seventy percent and above similarities of learning issues relating to predetermined faculty objectives were generated against nine of 16 triggers. All 8-textual formatted triggers showed >60% similarities while three (3/8) illustrated triggers showed <60% similarities, although highest (78.8%) similarities was generated against illustrated trigger (Table-II). Textual triggers designed with unambiguous languages which may lead students to generate learning issues more-matched with faculty objectives. Angeli^10^ reported high school teachers faced challenges in trigger design for PBL. Careful thought is needed in designing instruction as different types of instruction are meant for different learning outcomes.^11^ Formats whether textual or illustrated, the quality depends on trigger structure, clarity, difficulty, familiarity, relevance and motivation to tackle the problem. Triggers should serve, to engage students, spark discussions, encourage collaboration, promote self-directed learning and lead acquisition of relevant content knowledge.^12^ Size of triggers is a matter; triggers could be ineffective if the contents are insufficient, excessive, off-topic, over or below learners’ abilities and include unintended ambiguous information.^13^ Schmidt & Moust^14^ opined that, a trigger should broadcast only a limited number of issues as learners cannot grip too many topics at the same time; within one trigger, two-three major issues are sufficient to keep the students busy. Huge content will make learners’ cognitive system overloaded and make learning complicated. PBL triggers needs to lead questioning of learners to explore the information.^15^ Dolmans et al.^16^ prescribed seven principles of effective trigger design which includes: trigger should stimulate real-life, lead elaboration, encourage knowledge integration, encourage self-directed learning, fit-in with students’ prior knowledge, is of students’ interest, be of adequate level of complexity, structuredness and reflect faculty objectives.

This study is important as it provides an overview of PBL trigger characteristics at CFS IIUM. Lot of dedication will be required by lecturer to understand the characteristics of effective PBL trigger and face the challenges in designing triggers that leads to preparing the students with analytical and lifelong learning skills. Perspectives are changing as time is changing; teachers’ role is changing from deliverer to a designer of learning experiences.^17^ More efforts will be needed to know and master the skills.^18^ It is the commitment of the university to update curriculum and utilize and mobilize the appropriate resources for appropriate tasks.^19^ Proper teacher development training at regular interval is necessary to enhance PBL trigger design skill that leads to generate learning issues by the student correlating with predetermined faculty objectives.

## CONCLUSION

An average of 68% students’ generated learning issues with a variation from 58%-79% were matched with predetermined faculty objectives. The less the predetermined faculty objectives, the more were the matching. Seventy percent and above similarities were achieved from discussion of nine out of 16 PBL triggers. Designing an effective PBL trigger is a challenging task. Whatever the trigger formats, it is the designing of PBL trigger considering influential variables that influences higher outcomes. Principles of effective trigger design should be kept in mind. Educational institution should emphasize on training needs of faculty at regular interval in order to develop and re-in force teachers’ skills in PBL trigger design and thus promote a sustainable educational and finally organizational development.
